# The follicular-phase depot GnRH agonist protocol results in a higher live birth rate without discernible differences in luteal function and child health versus the daily mid-luteal GnRH agonist protocol: a single-centre, retrospective, propensity score matched cohort study

**DOI:** 10.1186/s12958-022-01014-0

**Published:** 2022-09-19

**Authors:** Ying Zhang, Wenxian Zhao, Yifan Han, Xin Chen, Shaoyuan Xu, Yueyue Hu, Honglu Diao, Changjun Zhang

**Affiliations:** 1 Reproductive Medicine Center, Renmin Hospital, Hubei University of Medicine, Shiyan, People’s Republic of China; 2 Hubei Clinical Research Center for Reproductive Medicine, Shiyan, People’s Republic of China; 3 Biomedical Engineering College, Hubei University of Medicine, Shiyan, People’s Republic of China; 4 Biomedical Research Institute, Hubei University of Medicine, Shiyan, People’s Republic of China; 5 Hubei Key Laboratory of Embryonic Stem Cell Research, Hubei University of Medicine, Shiyan, People’s Republic of China

**Keywords:** Live birth rate, Luteal support, Neonatal outcomes, Depot GnRH agonist protocol, Long GnRH agonist protocol

## Abstract

**Background:**

The gonadotropin-releasing hormone agonist (GnRH-a) has been used in in vitro fertilization/intracytoplasmic sperm injection (IVF/ICSI) cycles for a long time. This paper evaluates the efficacy and safety of two commonly used protocols (follicular-phase depot GnRH-a protocol and daily mid-luteal long GnRH-a protocol) in normal responders undergoing IVF/ICSI using propensity score matching (PSM) analysis.

**Methods:**

A total of 6,816 infertile women treated within the period from January 2016 to September 2020 were stratified into cohorts. A total of 2,851 patients received the long-acting group (depot GnRH-a protocol), and 1,193 used the short-acting group (long GnRH-a protocol) after the data-selection process. PSM was utilized for sampling by up to 1:1 nearest neighbour matching to adjust the numerical difference and balance the confounders between groups. The primary outcome was the live birth rate (LBR). Multivariable logistic analysis was used to evaluate the difference between these two protocols in relation to the LBR.

**Result(s):**

In this study, 1:1 propensity score matching was performed to create a perfect match of 964 patients in each group. After matching, the blastocyst formation rates, oestradiol (E_2_) value on Day hCG + 9, progesterone (P) value on Day hCG + 9, implantation rates, clinical pregnancy rates, and LBR were more favourable in the depot GnRH-a protocol than in the long GnRH-a protocol (*P* < 0.05). However, the moderate or severe OHSS rates were higher in the depot group than in the long group (*P* < 0.001). There were no significant differences in endometrial thickness, luteal support medication, early pregnancy loss rates, mid- and late-term pregnancy loss rates, or foetal malformation rates between the two protocols.

**Conclusion(s):**

Compared with the daily short-acting GnRH agonist protocol, the follicular-phase depot GnRH-a protocol might improve LBRs in normogonadotropic women without discernible differences in luteal function and child health.

**Supplementary Information:**

The online version contains supplementary material available at 10.1186/s12958-022-01014-0.

## Background

The gonadotropin-releasing hormone agonist GnRH-a has been used for preventing premature luteinization during controlled ovarian stimulation (COS) treatment for many years. Due to pituitary desensitization, lower spontaneous ovulation rates and higher pregnancy rates have been successfully achieved. In retrospect, there are two patterns of GnRH-a administration for achieving the effect of pituitary downregulation: one consisting of a short-acting daily low dose of GnRH-a (0.05–0.1 mg) committed in the luteal phase, which is called the “long protocol” and the “standard COS protocol”, and another consisting of a long-acting depot high dose of GnRH-a analogues with different doses (1.0–3.75 mg) and various durations (14–28 d) administered in the early follicular phase or luteal phase [1–3]. Although GnRH-a has been used in in vitro fertilization/intracytoplasmic sperm injection (IVF/ICSI) cycles for a long time, there is still controversy as to which form of GnRH-a administration is more effective.

The introduction of the depot GnRH-a protocol in COS opened up a new approach towards more “friendly IVF”, with the advantage of being more convenient by eliminating the need for multiple injections [4]. The standard full dose of depot GnRH-a is 3.75 mg. A previous study demonstrated that a full-dose depot GnRH-a injection was sufficient to maintain luteinizing hormone (LH) suppression until week 8 after the injection, oestradiol (E_2_) secretion started to be restored in the course of weeks 7–8, and suppression of pituitary and ovarian function appeared to be continued until week 8 after the injection [5]. Given that full-dose GnRH-a may excessively inhibit the pituitary gland and ovary, some of the early studies focused on reducing the GnRH-a dose to reduce the dosage and duration of gonadotropin (Gn) [6–8]. It was noted that an essential part of full-dose GnRH-a is involved in the improvement of follicular synchronization and endometrial receptivity. As a consequence, this prolonged downregulation before controlled ovarian hyperstimulation (COH) and embryo transfer (ET) might be acceptable in patients with endometriosis [9], adenomyosis [10, 11], and a general cohort [12, 13]. However, several studies have been performed to compare outcomes between the depot GnRH-a protocol and long protocol, with different conclusions presented regarding Gn doses, the duration of stimulation, the number of retrieved oocytes, fertilization rates, and pregnancy rates [1, 6, 13–19]. In addition, it may be more suitable and comprehensive to estimate the availability of the full-dose GnRH-a protocol after a full consideration of factors such as possible effects on oocytes or embryos, the luteal phase, and teratogenic effects.

Therefore, in this study, we report a comparison of the efficacy and safety of the two commonly used protocols (depot GnRH-a and long GnRH-a) on the laboratory and clinical outcomes after fresh ET in normal responders in a propensity score matching (PSM) retrospective cohort study, and we provide some suggestions for clinical practice.

## Methods

### Participants

We conducted a hospital-based cohort study. This investigation was performed in accordance with the principles of the Declaration of Helsinki and was approved by the Ethics Committee of Renmin Hospital, Hubei Medical University. Anonymous data were collected from the Reproductive Medicine Center, Renmin Hospital, Hubei University of Medicine, between January 2016 and September 2020.

Patients who received the early-follicle-phase depot GnRH-a and long GnRH-a protocols were included. Patients were selected if they met all the following inclusion criteria: women with regular menstrual cycles ranging from 25 to 35 days; aged < 40 years; body mass index (BMI) 18–28 kg/m^2^; normal basal serum follicle-stimulating hormone (FSH) (< 10 mIU/ml), and anti-Müllerian hormone (AMH) (≥ 1.1 ng/ml) levels determined on Days 2–3 of the cycle prior to COH. The exclusion criteria were as follows: patients with metabolic disorders, pelvic tuberculosis, congenital uterine malformations, chromosomal abnormalities or single-gene disorders, cardiovascular diseases, and tumours. We followed women by telephone until they had pregnancy outcomes.

### Study procedures

In the early-follicle-phase depot GnRH-a protocol (depot protocol), the patients received a single intramuscular injection of 3.75 mg long-acting triptorelin acetate (Decapeptyl; Ferring, SaintPrex, Switzerland) on Day 2 or 3 of the cycle. After 30–42 days of downregulation, an ultrasound scan and serum concentration tests were confirmed, and the criteria were as follows: endometrial thickness ≤ 5 mm; follicles 5–7 mm; serum concentration of E_2_ < 50 pg/ml; progesterone (*P*) < 1 ng/ml; and LH < 1 mIU/ml. Recombinant LH (Luveris; Merck Serono) (75 IU per day) was added in the mid- and late-follicular stages to promote follicular development when the serum LH level was below 1.2 mIU/ml.

**Fig. 1:**
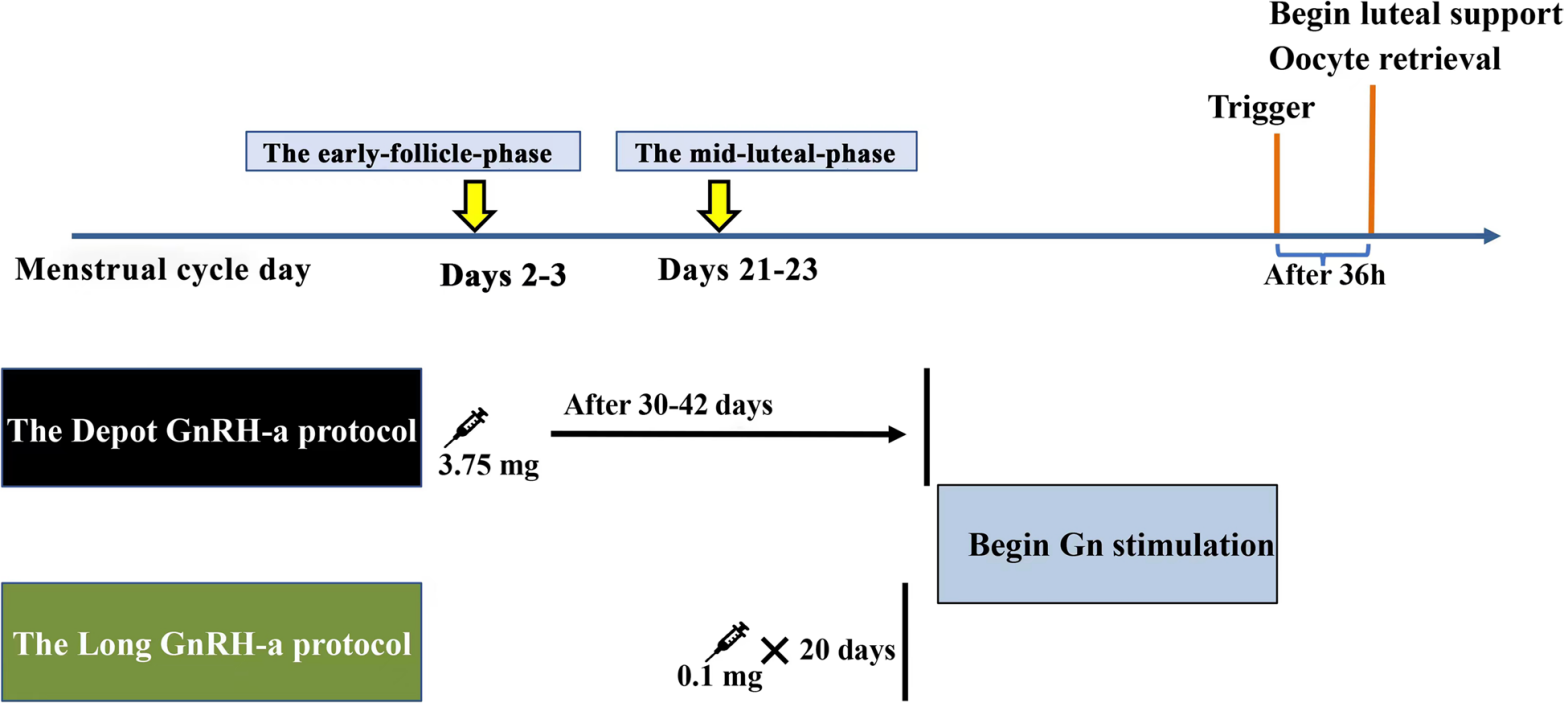
The diagram of the two GnRH-a protocols

In the long GnRH-a protocol (long protocol), a daily injection of 0.1 mg triptorelin acetate s.c. (Decapeptyl; Ferring, SaintPrex, Switzerland) in the midluteal phase (Days 21–23) of the menstrual cycle preceding treatment was used for pituitary downregulation. Downregulation was confirmed after 20 days following the same criteria described in the depot protocol (Fig. 1).

In all treatment protocols followed by Gn stimulation, the doses of recombinant FSH (Gonal-f, Merck Serono, Germany) and urinary human menopausal gonadotropin (HMG, Livzon Pharmaceutical, China) were adjusted according to the growth trend of follicles and serum hormone changes (150–450 IU per day). Recombinant human chorionic gonadotrophin (hCG) (Merck Serono, Italy) at a dose of 250 µg and urinary hCG (Livzon Pharmaceutical, China) at a dose of 1,000–2,000 IU were administered to trigger oocyte maturation when two or more follicles reached preovulatory size (18–22 mm). Oocyte retrieval was performed 36 h after the trigger. All oocytes were inseminated by IVF or ICSI according to the laboratory’s routine insemination procedures. Embryo scoring was conducted based on morphologic criteria; good-quality embryos were defined as 6–8 cells with less than 20% fragments. ET was performed on Day 3 or Day 5 using a soft catheter with transabdominal ultrasound guidance. After oocyte retrieval, luteal-phase support was initiated and continued daily until 3 months of gestation with the daily application of 90 mg vaginal progesterone gel (Crinone; Merck Serono) and either 10 mg twice or three times daily oral dydrogesterone (Duphaston, Abbott, USA), 2 mg twice daily oestradiol valerate tablets (Progynova, Berlin, Germany), or 1 mg:10 mg daily vaginal oestradiol and dydrogesterone tablets (Femoston, Abbott, USA). The good spare embryos were cryopreserved through a vitrification protocol. Fresh ET cancellation and freeze-all strategies were implemented in cases of high progesterone concentrations on hCG day (> 2 ng/ml) or to prevent ovarian hyperstimulation syndrome (OHSS).

Substantial quantities of steroid hormones (P and E_2_) were produced by the corpus luteum for the establishment and maintenance of pregnancy; thus, we measured serum P and E_2_ levels, which represent luteal function. According to the study published by Shahar Kol [20], luteolysis starts 48 h post-oocyte retrieval (approximately hCG + 3.5 days) in most patients, and a drop in the P level from Day 5 to Day 7 post hCG trigger was associated with a significantly lower ongoing pregnancy rate. Therefore, we chose the luteal support medication according to the P and E_2_ levels of hCG + 4 days: when the E_2_ level was more than 1000 pg/ml and the P level was more than 100 ng/ml, Crinone was used alone; when the E_2_ level was more than 1000 pg/ml and the P level was 50–100 ng/ml, both Crinone and dydrogesterone were used; when the E_2_ level was less than 1000 pg/ml and the P level was 50–100 ng/ml, Crinone and dydrogesterone plus Progynova were used; and when the E_2_ level was less than 500 pg/ml and the P level was less than 50 ng/ml, Crinone, dydrogesterone, Progynova and Femoston were used.

### Outcome parameters

In this study, the ovarian stimulation characteristics of the patients, including the dosage of Gn, duration of Gn, endometrial thickness on the hCG day, moderate or severe OHSS rates, and luteal support medication, were evaluated. The parameters of oocytes and embryos and pregnancy outcomes, including the number of oocytes retrieved, biochemical pregnancy rate (PR), clinical PR and pregnancy loss rate, were also recorded. Moderate or severe OHSS was diagnosed in women who fulfilled more than one of the following criteria: clinical ascites, hydrothorax, or dyspnoea (exertional or at rest) [21]. Biochemical pregnancy was defined as hCG > 10 mIU/ml two weeks after ET. Clinical pregnancy was defined as an intrauterine gestational sac identified by ultrasonography 30 days after ET. Early pregnancy loss was defined as spontaneous pregnancy loss before 12 weeks. Live birth was considered when a living foetus was born after 28 weeks of pregnancy.

The primary outcome was the LBR. The secondary outcomes were implantation, clinical pregnancy, pregnancy loss, moderate or severe OHSS rates, endometrial thickness on the hCG day, luteal support medication, and neonatal outcomes.

### Statistical methods

Data are presented as the mean ± standard deviation (SD) or as the frequency and percentage (for categorical variables). Continuous variables were analysed with an independent-sample Student *t* test. Pregnancy outcomes were compared with the *x*^*2*^ test where appropriate. A multivariable logistic regression analysis was performed to assess significant relationships between two different COS protocols and pregnancy outcomes.

Given the differences in the baseline characteristics between eligible participants in the two groups, PSM was used to identify a cohort of patients with similar baseline characteristics. The propensity score for using different COS protocols was developed on the basis of the following variables: female age, BMI, AFC, AMH, infertility duration, infertility type, infertility factors, number of transferred embryos and embryo transfer day. Matching was performed with the use of a 1:1 matching protocol without replacement (greedy-matching algorithm), with a caliper width equal to 0.01 of the standard deviation of the logit of the propensity score. Covariate balance was assessed in these matched participants by checking standardized differences between the two groups (Y = Depot GnRH-a and Y = Long GnRH-a); we considered a covariate to be well balanced if the standardized difference was less than 0.1. After PSM, between-group differences for all covariates involved in matching were eliminated. Finally, conditional logistic regression was employed to determine the association between these two protocols and pregnancy outcomes in a post-PSM matched population.

All statistical analyses were performed using the Statistical Package for the Social Sciences (SPSS) version 22.0 and EmpowerStats (http://www.empowerstats.com; X&Y Solutions, Inc., Boston, MA, USA). Statistical significance was accepted as a two-sided *P* value < 0.05. Graphs were generated by using GraphPad Prism version 5.0 (GraphPad Software).

## Results

### Study population

The data selection process is illustrated in Supplemental Fig. 1. From the initial cohort of 6,816 IVF/ICSI cycles, 1,580 cycles were excluded from the analysis for the following reasons: female age > 40 years, basal FSH > 10 mIU/ml, AMH < 1.1 ng/ml, antral follicle count (AFC) < 7, 18 ≤ BMI ≤ 28 kg/m^2^, uterine anomalies, and polycystic ovary syndrome (PCOS). All the patients had complete downregulation, COH, and ET processes except for the following: 21 women had cancelled downregulation, five women had cancelled COH processes because of follicular dysplasia, 78 women had cancelled COH processes because of the strict control of human activities due to the coronavirus (COVID-19) outbreak, 29 women had cancelled fertilization for oocyte cryopreservation, 10 woman had cancelled ET because of complications after oocytes were retrieved, 15 woman had cancelled ET because of abnormal hormone levels, 162 woman had cancelled ET due to endometrial reasons, 188 woman had cancelled ET due to the adoption of preimplantation genetic testing (PGT), and 684 woman had cancelled ET because of a high risk of OHSS.

After these exclusions, the eligible cohort included 1,193 women using the long GnRH-a protocol, 2,851 women using the depot GnRH-a protocol, and 964 patients in each group when PSM was performed (Supplemental Table 1). The standardized differences in all covariates involved in matching were less than 0.1. There were no statistically significant differences in female age, BMI, AFC, AMH, infertility duration, infertility type, infertility factors, number of transferred embryos and embryo transfer day in the two groups (*P* > 0.05) (but *P* = 0.035 for AMH) (Table 1).

**Table 1:** Basic characteristics of the two GnRH-a protocols before and after propensity score matching

	Before propensity matching			After propensity matching			
	Long GnRH-a	Depot GnRH-a	*P*-value	Long GnRH-a	Depot GnRH-a	Standardized diff	*P*-value
No. of cycles	1193	2851		964	964		
Female Age (years)	30.03 (3.77)	29.98 (3.73)	0.689	30.07 (3.79)	29.95 (3.73)	0.032	0.482
BMI (kg/m^2^)	22.34 (2.60)	22.67 (2.67)	< 0.001	22.27 (2.59)	22.25 (2.57)	0.007	0.878
AFC	14.26 (4.99)	15.37 (5.31)	< 0.001	14.17 (4.99)	14.52 (5.07)	0.068	0.135
AMH (ng/ml)	4.42 (2.66)	5.35 (3.02)	< 0.001	4.36 (2.49)	4.59 (2.42)	0.096	0.035
Infertility duration (years)	3.68 (2.70)	3.49 (2.64)	0.037	3.65 (2.67)	3.63 (2.76)	0.007	0.872
Infertility type, n (%)			0.018			0.048	0.295
Primary	582/1193 (48.78)	1507/2851 (52.86)		464/964 (48.13)	487/964 (50.52)		
Secondary	611/1193 (51.22)	1344/2851 (47.14)		500/964 (51.87)	477/964 (49.48)		
Fertilization method, n (%)			0.762			0.029	0.528
IVF	936/1193 (78.46)	2249/2851 (78.88)		770/964 (79.88)	781/964 (81.02)		
ICSI	257/1193 (21.54)	602/2851 (21.12)		194/964 (20.12)	183/964 (18.98)		
Infertility factors, n (%)			0.002				0.634
Pelvic and tubal factors	802/1193 (67.23)	1780/2851 (62.43)		663/964 (68.78)	653/964 (67.74)	0.022	
Ovulation disorder	88/1193 (7.38)	288/2851 (10.10)		73/964 (7.57)	69/964 (7.16)	0.016	
Endometriosis	29/1193 (2.43)	113/2851 (3.96)		22/964 (2.28)	33/964 (3.42)	0.069	
Male factor	209/1193 (17.52)	487/2851 (17.08)		161/964 (16.70)	160/964 (16.60)	0.003	
Unexplained	65/1193 (5.44)	183//2851 (6.43)		45/964 (4.67)	49/964 (5.08)	0.019	

Date: mean (SD) or (%) (no./total no.)

*GnRH-a* gonadotropin-releasing hormone agonist, *BMI* body mass index, *AFC* antral follicular count, *AMH* anti-Müllerian hormone, *IVF *in vitro fertilization, *ICSI*, intracytoplasmic sperm injection

### Ovarian stimulation characteristics

The ovarian stimulation characteristics of the two groups are given in Table 2. After PSM, there were significant differences in FSH, LH, and E_2_ values on the Gn starting day; FSH, LH, E_2_, and P values on the hCG day; the duration of Gn, moderate or severe OHSS rates, fertilization rates, cleavage rates, number of embryos obtained, good-quality embryo rates and blastocyst formation rates (*P* < 0.05). However, there were no statistically significant differences between the two groups in terms of the P values on the Gn starting day, endometrial thickness on the hCG day, dosage of Gn, number of oocytes retrieved, number of mature oocytes, number of transferred embryos and number of embryo transfer days (*P* > 0.05).

**Table 2:** Ovarian stimulation characteristics according to the two GnRH-a protocols before and after propensity score matching

	Before propensity matching			After propensity matching		
	Long GnRH-a	Depot GnRH-a	*P*-value	Long GnRH-a	Depot GnRH-a	*P*-value
FSH value on starting day (mIU/mL)	4.57 (1.98)	2.84 (1.97)	< 0.001	4.54 (2.02)	2.81 (1.96)	< 0.001
LH value on starting day (mIU/mL)	2.43 (1.38)	0.86 (0.52)	< 0.001	2.43 (1.40)	0.88 (0.55)	< 0.001
E_2_ value on starting day (pg/ml)	31.82 (38.17)	21.23 (20.94)	< 0.001	30.35 (35.26)	21.49 (18.51)	< 0.001
P value on starting day (ng/ml)	0.53 (1.57)	0.42 (1.14)	0.017	0.51 (1.45)	0.49 (1.91)	0.785
FSH value on hCG day (mIU/mL)	16.49 (6.62)	17.00 (5.75)	0.016	16.52 (6.70)	17.57 (5.98)	< 0.001
LH value on hCG day (mIU/mL)	2.96 (1.72)	1.07 (0.76)	< 0.001	2.91 (1.68)	1.12 (0.71)	< 0.001
E_2_ value on hCG day (pg/ml)	3734.31 (1458.32)	2555.67 (1094.42)	< 0.001	3737.01 (1489.16)	2536.74 (1094.60)	< 0.001
P value on hCG day (ng/ml)	1.03 (0.45)	0.87 (0.44)	< 0.001	1.03 (0.46)	0.88 (0.42)	< 0.001
Endometrial thickness on hCG day(mm)	11.71 (2.69)	11.82 (2.73)	0.260	11.75 (2.69)	11.82 (2.74)	0.601
Dosage of Gn (IU)	2397.16 (908.41)	2433.40 (850.62)	0.226	2401.94 (911.91)	2416.23 (810.19)	0.716
Duration of Gn (days)	10.25 (1.34)	11.77 (1.72)	< 0.001	10.28 (1.34)	11.76 (1.69)	< 0.001
Moderate or severe OHSS rates, n (%)	4/1193 (0.34)	34/2851 (1.19)	< 0.001	2/964 (0.21)	7/964 (0.73)	< 0.001
No. of oocytes retrieved	9.38 (2.77)	9.67 (2.70)	0.002	9.29 (2.79)	9.41 (2.76)	0.345
No. of mature oocytes	8.64 (2.71)	8.97 (2.63)	< 0.001	8.58 (2.71)	8.77 (2.69)	0.136
Fertilization rate (2PN) (%)	88.21 (12.05)	90.32 (10.92)	< 0.001	87.97 (12.19)	90.20 (11.02)	< 0.001
Cleavage rate (%)	98.09 (5.80)	98.62 (4.75)	0.003	98.06 (5.75)	98.85 (4.07)	< 0.001
No. of embryos obtained	3.83 (1.37)	3.99 (1.37)	< 0.001	3.84 (1.38)	3.98 (1.36)	0.026
good-quality embryo rate (%)	66.91 (29.17)	70.82 (27.13)	< 0.001	67.79 (29.25)	72.17 (26.56)	< 0.001
Blastocyst formation rate (%)	56.57(42.88)	65.42(41.28)	< 0.001	56.19 (42.66)	65.37 (41.22)	< 0.001
No. of transferred embryos, n (%)			0.298			0.568
1	397/1193 (33.28)	901/2851 (31.60)		338/964 (35.06)	350/964 (36.31)	
2	796/1193 (66.72)	1950/2851 (68.40)		626/964 (64.94)	614/964 (63.69)	
Embryo transfer day, n (%)			< 0.001			0.274
Day 3	652/1193 (54.65)	1385/2851 (48.58)		520/964 (53.94)	496/964 (51.45)	
Day 5	541/1193 (45.35)	1466/2851 (51.42)		444/964 (46.06)	468/964 (48.55)	

Date: mean (SD) or (%) (no./total no.)

*GnRH-a* gonadotropin-releasing hormone agonist, *FSH* follicle-stimulating hormone, *LH* luteinizing hormone, *E*_*2*_ oestradiol, *P* progesterone, *hCG* human chorionic gonadotrophin, *Gn* Gonadotropin, *OHSS* ovarian hyperstimulation syndrome, *PN* pronuclear number

### Hormone profile, luteal support, and pregnancy outcomes

For hormone levels after oocytes were retrieved, there were no statistically significant differences in E_2_ and P levels of the two cohorts on Day hCG + 4 (*P* > 0.05), but there were significant differences on Day hCG + 9 (*P* < 0.001) (Table 3). There were no statistically significant differences between the two groups in luteal-phase support methods (*P* > 0.05) (Table 3). The implantation rates, biochemical PR, clinical pregnancy rates (CPR), and LBR were significantly higher in the depot GnRH-a group than in the long GnRH-a group (*P* < 0.05) (Table 3, Supplemental Table 2, Supplemental Fig. 2). There was no statistically significant difference between the two groups in ectopic pregnancy rates, early pregnancy loss rates (EPLRs), mid- and late-term pregnancy loss rates, or preterm birth rates (*P* > 0.05) (Table 3).

**Table 3:** Hormone profile, luteal support, and pregnancy outcomes according to the two GnRH-a protocols before and after propensity score matching

	Before propensity matching			After propensity matching		
	Long GnRH-a	Depot GnRH-a	*P*-value	Long GnRH-a	Depot GnRH-a	*P*-value
E_2_ value on hCG + 4 day (pg/ml)	1316.43 (569.82)	1292.35 (591.67)	0.252	1308.32 (571.99)	1277.97 (555.17)	0.267
P value on hCG + 4 day (ng/ml)	182.55 (68.12)	194.24 (71.28)	0.057	183.92 (67.74)	186.01(71.13)	0.528
E_2_ value on hCG + 9 day (pg/ml)	1301.87 (1095.22)	1663.89(1184.46)	< 0.001	1304.89(1109.56)	1699.51(1183.88)	< 0.001
P value on hCG + 9 day (ng/ml)	78.57 (67.71)	100.40 (91.68)	< 0.001	80.17 (69.03)	107.95 (96.44)	< 0.001
Luteal support, n (%)			0.111			0.057
C	133/1193 (11.15)	334/2851 (11.72)		90/964 (9.34)	108/964 (11.20)	
C + D	92/1193 (7.71)	238/2851 (8.35)		80/964 (8.30)	99/964 (10.27)	
C + D + P	703/1193 (58.93)	1741/2851 (61.07)		577/964 (59.85)	579/964 (60.06)	
C + D + P + F	265/1193 (22.21)	538/2851 (18.86)		217/964 (22.51)	178/964 (18.46)	
Implantation rate (%)	47.05 (43.70)	52.49 (44.13)	< 0.001	47.44 (43.81)	52.26 (44.60)	0.017
Biochemical pregnancy rate (%)	769/1193 (64.46)	1976/2851(69.31)	0.003	619/964 (64.21)	665/964 (68.98)	0.026
Clinical pregnancy rate (%)	696/1193 (58.34)	1802/2851 (63.21)	0.004	552/964 (57.26)	600/964 (62.24)	0.026
Ectopic pregnancy rate (%)	10/696 (1.44)	25/1802 (1.39)	0.469	9/552 (1.63)	13/600 (2.17)	0.667
No. of fetuses in pregnancy, n (%)			0.011			0.033
Single	520/696 (74.71)	1237/1802 (68.65)		419/552 (75.91)	422/600 (70.33)	
Twins	176/696 (25.29)	565/1802 (31.35)		133/552 (24.09)	178/600 (29.67)	
Early pregnancy loss rate (%)	82/696 (11.78)	209/1802 (11.60)	0.469	63/552 (11.41)	61/600 (10.17)	0.667
Mid- and late-term pregnancy loss rate (%)	23/696 (2.44)	51/1802 (2.83)	0.469	13/552 (2.36)	15/600 (2.50)	0.667
Preterm birth rate (%)	91/696 (13.07)	285/1802 (15.82)	0.093	66/552 (11.96)	87/600 (14.50)	0.214
Live birth rate (%)	581/1193 (48.70)	1517/2851 (53.21)	0.009	467/964 (48.44)	511/964 (53.01)	0.045

Date: mean (SD) or (%) (no./total no.)

*GnRH-a* gonadotropin-releasing hormone agonist, *E*_*2*_ oestradiol, *hCG* human chorionic gonadotrophin, *P* progesterone, *C* Crinone, *C* + *D* Crinone + Dydrogestrone, *C* + *D* + *P* Crinone + Dydrogestrone + Progynova, *C* + *D* + *P* + *F* Crinone + Dydrogestrone + Progynova + Femostone

### Neonatal outcomes

Descriptive statistics for the neonatal outcomes are summarized in Table 4. The birth weights differed significantly between the two groups (*P* < 0.01). However, no significant association was observed between gestational weeks, number of singleton live births, birth sex ratio, and rates of congenital malformation in the two groups (Table 4).

**Table 4:** Neonatal outcomes according to the two GnRH-a protocols before and after propensity score matching

	Before propensity matching			After propensity matching		
	Long GnRH-a	Depot GnRH-a	*P*-value	Long GnRH-a	Depot GnRH-a	*P*-value
Gestational weeks	38.08 (1.89)	37.93 (2.05)	0.121	38.17 (1.76)	38.03(1.98)	0.270
Birth weights(g)	3133.28 (572.87)	3042.07 (594.01)	0.002	3165.93 (564.08)	3059.98 (587.38)	0.004
Fetuses delivered, n (%)			0.002			0.056
Single	449/581 (77.28)	1069/1517 (70.47)		367/467 (78.59)	374/511 (73.19)	
Twins	132/581 (22.72)	448/1517 (29.53)		100/467 (21.41)	137/511 (26.81)	
Fetus's sex, n (%)			0.026			0.371
A boy	251/581 (43.20)	583/1517 (38.43)		198/467 (41.40)	201/511 (39.33)	
A girl	198/581 (34.08)	486/1517 (32.04)		169/467 (36.19)	173/511 (33.86)	
Two boys	40/581 (6.88)	143/1517 (9.43)		31/467 (6.64)	40/511 (7.83)	
Two girls	24/581 (4.13)	97/1517(6.39)		22/467 (4.71)	35/511 (6.85)	
A boy and a girl	68/581 (11.70)	208/1517 (13.71)		47/467 (10.06)	62/511 (12.13)	
Fetal malformation rate (%)	5/581 (0.86)	16/1517 (1.05)	0.694	4/467 (0.86)	9/511 (1.76)	0.147

Date: mean (SD) or (%) (no./total no.)

*GnRH-a* gonadotropin-releasing hormone agonist

## Discussion

In this large-scale PSM retrospective cohort study of 6,816 women undergoing IVF/ICSI, there was a significantly higher LBR in the depot GnRH-a protocol than in the long GnRH-a protocol. This study is unique in that the PSM method was used, and the corpus luteum function and health of the offspring were estimated, thereby fully exploring the advantages and disadvantages of prolonged downregulation.

We found that there was no difference in hormonal suppression after downregulation between the depot GnRH-a protocol and the long GnRH-a protocol. This finding is aligned with a previously published study demonstrating that rapid, profound, and sustained suppression of pituitary and ovarian function was achieved after Day 21 of administration by both protocols [14]. However, differences were found in the time of resumption of pituitary activity, which takes place 7 days after the discontinuation of the daily form and approximately 2 months after discontinuation of the depot form [22]. These findings suggest a more profound blockage of ovarian function and a more prolonged duration of action by depot GnRH-a administration. Three types of problems may be related to this point: possible detrimental effects on oocytes or embryos, possible unfavourable effects on the luteal phase, and possible teratogenic effects.

It is not clear whether oocytes or embryos are defective under the effects of long-acting GnRH-a in IVF cycles. In the process of downregulation, GnRH-a inhibits FSH and LH to different degrees, mainly inhibiting LH; therefore, there may be insufficient LH [23]. LH plays two roles in the process of follicular development. In the early follicular stage, it stimulates theca cells to produce androgens; in the middle follicular stage, it stimulates granulosa cells to produce various cytokines to promote the growth of granulosa cells per se and then promote the maturation of oocytes. The LH level between the lowest threshold and the upper limit is called the LH window [24]. Appropriate follicular development, maturation, and steroid synthesis require a minimum threshold level of LH, but the amount required is small; as long as 1% receptor is occupied, this is enough to maintain steroid synthesis. The lower limit of the LH threshold ranges between 0.5 and 1.2 mIU/mL [25]. Consistent with previous reports, this study demonstrated that the serum LH level of the depot GnRH-a protocol on the Gn starting day was lower than that of the long GnRH-a protocol (0.88 ± 0.55 mIU/ml vs. 2.43 ± 1.40 mIU/ml, *P* < 0.001). In addition, a dynamic decrease in serum LH levels during the early to mid-follicular stage was suggested as an indicator of LH deficiency, causing a reduced live birth rate [25]. A multicentre prospective randomized controlled trial (RCT) reported that the therapeutic benefit of exogenous LH at a daily dose of 75 IU is only observed when endogenous serum LH is below 1.2 mIU/ml [26]. LH supplementation seems to have added value for pregnancy achievement in women with a poor ovarian response and in women more than 35 years of age employing the GnRH-a protocol [25], which was also confirmed in our cohort of normal responders. These observations highlight the importance of adding a small amount of LH to overcome LH deficiency to ensure oocyte quality and therefore embryo quality.

Our observation is in agreement with the results obtained in previous studies, which showed that the E_2_ level of the depot protocol on the hCG trigger day was lower than that of the long protocol [13, 27]. An experiment on human cumulus cells (CC) indicated that serum E_2_ levels on the day of hCG administration are negatively correlated with luteinizing hormone and human chorionic gonadotrophin receptor (LH/hCGR) expression [28]. It has been shown that high LH/hCGR gene expression intensity is positively correlated with expanded CC morphology and oocyte maturation [29]. It was further proven in trophoblast cell spheroid/endometrial cell coculture experiments that excessive serum oestradiol levels following COS leads to changed steroid receptor expression [30], enhanced endometrial glandular cell apoptosis, and an altered implantation window [31], eventually leading to impaired endometrial receptivity and adverse reproductive outcomes. The above results confirm that relatively lower E_2_ levels after COS may result in good oocyte quality and are conducive to embryo implantation. As expected, we found that the blastocyst formation rate was significantly increased in the depot protocol compared with the long protocol (65.37 ± 41.22% vs. 56.19 ± 42.66%, respectively, *P* < 0.001), which has not been reported in previous reports; moreover, we did not identify the underlying molecular mechanism(s) linking the observed E_2_ level to embryo quality itself in a cause-and-effect relationship. Therefore, embryo quality should certainly still deserve full attention in our appraisal of depot GnRH-a downregulation linked to an increased pregnancy rate.

Adequate luteal function is essential for achieving and maintaining pregnancy. It is well established that luteal function is compromised by the direct effect of GnRH-a and low LH levels on the corpus luteum. It is not clear whether luteal function is more defective after depot GnRH-a downregulation than after short-acting GnRH-a downregulation in stimulated IVF cycles. Several aspects have been proposed to focus on this topic. First, whether depot GnRH-a downregulation changes endometrial thickness was initially neglected by earlier studies. A retrospective study documented that the depot protocol yielded significantly higher implantation rates and CPRs in patients with a medium (7–14 mm) (or especially, a thin [≤ 7 mm]) endometrium than the long protocol [17]. Another team maintained that the endometrial thickness and CPR were significantly greater in the long-acting GnRH-a group than in the short-acting GnRH-a group (12.05 ± 2.57 mm vs. 11.79 ± 2.54 mm; 68.22% vs. 58.86%, respectively; *P* < 0.001) after retrospective analysis [32]. Furthermore, a recent RCT concluded that there were no significant differences in endometrial thickness (12.29 ± 2.59 mm vs. 11.96 ± 2.62 mm, *P* = 0.33) but a significantly higher LBR in the depot protocol than in the long protocol (62.6% vs. 52.1%, *P* = 0.03) [13], which was also confirmed by our study. Therefore, endometrial thickness does not seem to be a strong parameter predicting success in IVF patients undergoing the depot protocol. Second, there is insufficient evidence regarding whether depot downregulation requires more luteal phase support (LPS) than the long protocol. P is the most pivotal hormone to maintain pregnancy, and E_2_ also plays an important role during this time. The hormone level and maintenance time are closely related to successful pregnancy by making the endometrium receptive to the embryo that will be implanted during the window of implantation [33]. It has been shown that the peak concentration of P occurs 4 days after oocyte pick-up (OPU + 4), followed by an average 35% decrease from OPU + 4 to OPU + 6 in patients undergoing IVF/ICSI without LPS [34]. Another study found that there were no differences in pregnancy outcomes between the depot protocol and the long protocol involving LPS by daily injections of 50 mg progesterone [22]. Our results do not confirm such data. In fact, we observed that the LBR was significantly higher in the depot GnRH-a group than in the long GnRH-a group, while the LPS methods were comparable in these two protocols. Specifically, our results identified differentiation of the E_2_ and P levels of the mid-luteal phase, which was significantly higher on Day hCG + 9 (OPU + 7) in the depot GnRH-a protocol than in the long GnRH-a protocol (1699.51 ± 1183.88 pg/ml vs. 1304.89 ± 1109.56 pg/ml; 107.95 ± 96.44 ng/ml vs. 80.17 ± 69.03 ng/ml, respectively, *P* < 0.001). Notably, the moderate or severe OHSS rates were evidently higher in the depot group than in the long group (0.73% vs. 0.21%, *P* < 0.001). All of this may be because pituitary resuscitation was in the luteal phase, and 6–7 weeks after the administration of depot GnRH-a, any detrimental effect on the luteal phase was thus increasingly weak; therefore, there may be no need for stronger corpus luteum support, and clinicians should be vigilant against the elevated risk of moderate or severe OHSS compared to that in the long protocol. To the best of our knowledge, this is the first contrastive analysis of LPS in these two protocols. Third, the EPLR is also a good index for the assessment of corpora luteal function. It should be noted that profound suppression of LH has previously been identified as a risk factor for EPLR [35]. However, this was not the case. Our data are in line with the results of previous studies, which showed similar miscarriage rates [4, 14, 22] in the depot and long protocols. Furthermore, in our data, not only the EPLR but also the mid- and late-term pregnancy loss rate (10.17% vs. 11.41%; 2.50% vs. 2.36%, *P* = 0.667) may well indicate comparable corpora luteal function in the two groups. Moreover, a recent study suggested that the abortion rate was significantly lower in the depot group than in the long group (5.54% vs. 9.37%, *P* < 0.01) [32]. Fourth, the effect of downregulation was confirmed by ultrasound scan and serum concentration tests, and follicular size of 5–7 mm were one of the criteria. In this study, if the follicular size was less than 5 mm, the ultrasound scan was repeated every 3 days until it was 5–7 mm. The starting doses were comparable between these two groups, but a lower total dose of Gn was found in the depot group, which may be because of delayed ovulation induction and the adjusted dose of Gn according to the growth trend of follicles and serum hormone changes in the mid- and late-follicular stages. The results of these studies, together with those presented here, support the conclusion that prolonged pituitary regulation seems to have no more adverse consequences on corpora luteal function.

Taking into account that there is a risk of exposure to GnRH-a in early pregnancy, teratogenicity after GnRH-a administration always needs to be considered for the health of offspring. To date, the neonatal health outcomes in the short-acting GnRH-a-exposed group were comparable to those of the control groups [36, 37]. However, there is insufficient evidence that the healthy outcomes of children after prolonged GnRH-a downregulation are better or worse than those of children with short-acting GnRH-a downregulation. By comparing the exposure days in the depot protocol in a retrospective analysis including 7007 patients, it was found that the LBR was noticeably higher in patients with more than 36 exposure days than in the less-than-30-day group and the 31–35-day group [38], but this is unfortunate due to the lack of offspring data. On the other hand, research on conventional IVF (including the short agonist and antagonist protocols) confirms that the proportion of small‐for‐gestational‐age cases was higher following conventional IVF than after natural cycle IVF (11.8% vs. 2.9%, *P* = 0.058), which is significantly positively associated with supraphysiological E_2_ levels on the hCG trigger day [39]. Inspired by these incomplete results using the different regimens, it is tempting to speculate that long-acting administration of GnRH-a may not have any teratogenic effect on offspring due to the characteristics of lower E_2_ levels, which surely is true of our study cohort and seems to be reassuring. This point requires further investigation.

The limitation of our study was that, due to its retrospective nature, even though patients were matched for propensity, individual differences may still have existed, possibly affecting the research results. Another limitation was that the dose of the hCG trigger differed between individuals, therefore differentially affecting the function of the corpus luteum. Our study did not have complete data on intrauterine growth retardation, the types and severity of birth defects. If these data become available, they would be more accurate in determining the effect of depot GnRH-a exposure on neonatal outcomes. In addition, a comparative study on the molecular mechanism of embryo quality and luteal function in these two protocols needs to be conducted further. Besides, the economic and time costs of the two protocols were not compared.

## Conclusion

In summary, the present study showed that the depot GnRH-a protocol appears to offer a significantly higher LBR than long GnRH-a protocols, and there is no increase in undesirable pregnancies. A possible explanation for the better results with the depot GnRH-a protocol may be a beneficial effect on hormone levels and embryo quality that is not necessarily harmful to luteal function. One should pay more attention to prevention of OHSS when using the depot GnRH-a protocol. More well-designed randomized controlled trials are needed to further compare the pros and cons of the two protocols.

## Supplementary Information

**Additional file 1: Supplemental Fig. 1.** Flow chart of patient inclusion/exclusion. GnRH-a, gonadotropin-releasing hormone agonist; n, number of participants; COH, controlled ovary hyperstimulation; COVID-19, coronavirus; ET, embryo transfer; PGT, preimplantation genetic diagnosis/screening; OHSS, ovarian hyperstimulation syndrome; FSH, follicle-stimulating hormone; AMH, anti-Müllerian hormone; AFC, antral follicle count; BMI, body mass index.

**Additional file 2: Supplemental Fig. 2.** Pregnancy outcomes according to the two GnRH-a protocols before and after propensity score matching.

**Additional file 3: Supplemental Table 1.** Propensity score parameter list.

**Additional file 3: Supplemental Table 2.** Comparison of the correlation between the two GnRH-a protocols and pregnancy outcomes using multivariable regression analysis and conditional logistic regression analysis before and after propensity score matching.

## Data Availability

All data presented in this study are available upon request upon contact with the corresponding author.

## Abbreviations

AFC: Antral follicle counts
AMH: Anti-Müllerian hormone
BMI: Body mass index
CC: Cumulus cells
COH: Controlled ovarian hyperstimulation
COS: Controlled ovarian stimulation
CPR: Clinical pregnancy rate
E_2_: Oestradiol
EPLR: Early pregnancy loss rate
ET: Embryo transfer
FSH: Follicle-stimulating hormone
Gn: Gonadotropin
GnRH-a: Gonadotropin-releasing hormone agonist
hCG: Human chorionic gonadotrophin
HMG: Human menopausal gonadotrophin
ICSI: Intracytoplasmic sperm injection
IVF: *In vitro* fertilization
LBR: Live birth rate
LH: Luteinizing hormone
LPS: Luteal phase support
OHSS: Ovarian hyperstimulation syndrome
P: Progesterone
PCOS: Polycystic ovary syndrome
PGT: Preimplantation genetic testing
PR: Pregnancy rate
PSM: Propensity score matching
SD: Standard deviation
