# Supplementary figures and images for: The follicular-phase depot GnRH agonist protocol results in a higher live birth rate without discernible differences in luteal function and child health versus the daily mid-luteal GnRH agonist protocol: a single-centre, retrospective, propensity score matched cohort study
Source: Reprod Biol Endocrinol. 2022 Sep 19;20:140. doi: 10.1186/s12958-022-01014-0 (PMC9483542; doi:10.1186/s12958-022-01014-0)

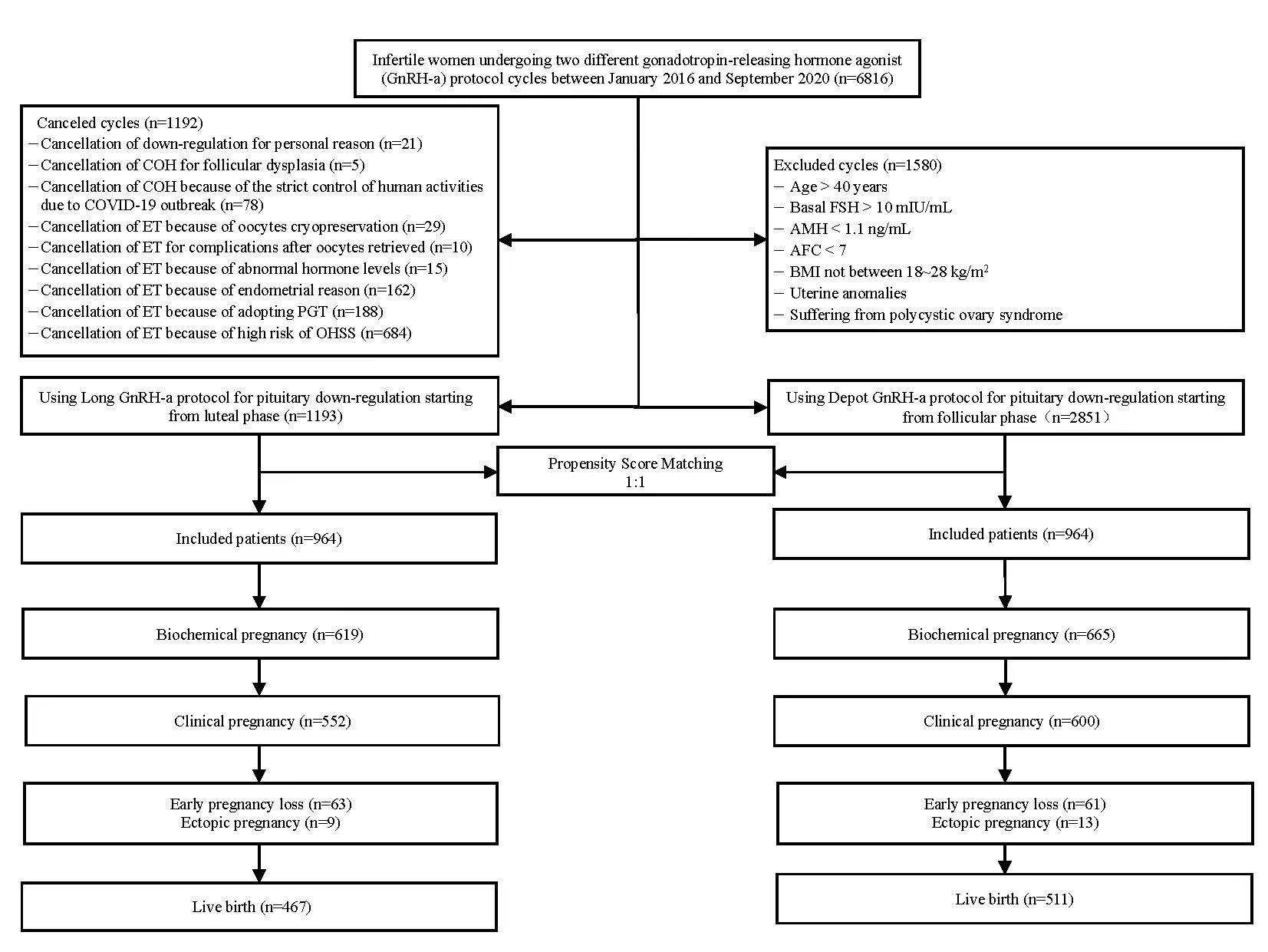

Supplement: Supplementary file 1 — Additional file 1: Supplemental Fig. 1. Flow chart of patient inclusion/exclusion. GnRH-a, gonadotropin-releasing hormone agonist; n, number of participants; COH, controlled ovary hyperstimulation; COVID-19, coronavirus; ET, embryo transfer; PGT, preimplantation genetic diagnosis/screening; OHSS, ovarian hyperstimulation syndrome; FSH, follicle-stimulating hormone; AMH, anti-Müllerian hormone; AFC, antral follicle count; BMI, body mass index. [file 12958_2022_1014_MOESM1_ESM.tiff]

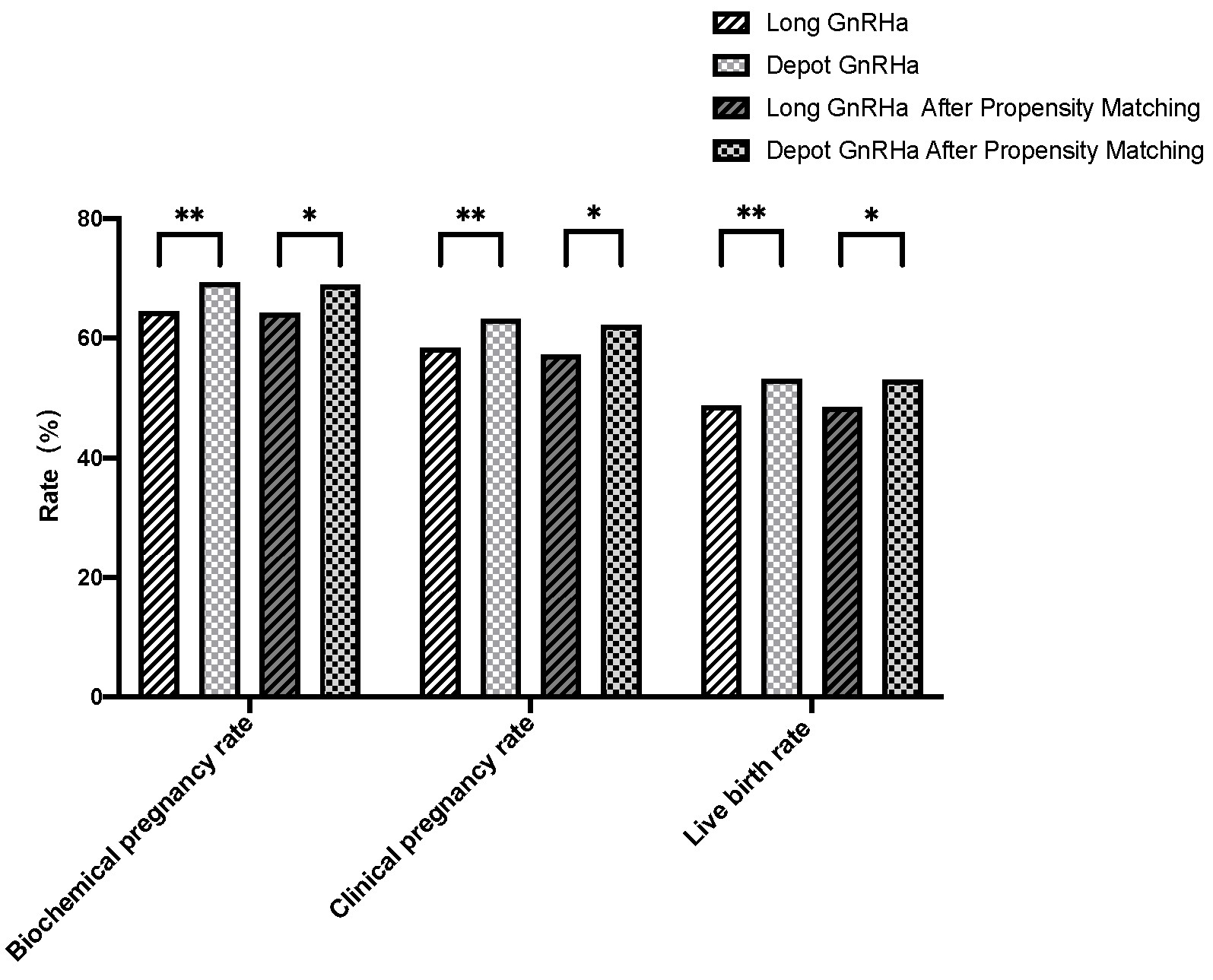

Supplement: Supplementary file 2 — Additional file 2: Supplemental Fig. 2. Pregnancy outcomes according to the two GnRH-a protocols before and after propensity score matching. [file 12958_2022_1014_MOESM2_ESM.tiff]
