# Supplementary material for: The follicular-phase depot GnRH agonist protocol results in a higher live birth rate without discernible differences in luteal function and child health versus the daily mid-luteal GnRH agonist protocol: a single-centre, retrospective, propensity score matched cohort study
Source: Reprod Biol Endocrinol. 2022 Sep 19;20:140. doi: 10.1186/s12958-022-01014-0 (PMC9483542; doi:10.1186/s12958-022-01014-0)
Supplement: Supplementary file 3 — Additional file 3: Supplemental Table 1. Propensity score parameter list. [file 12958_2022_1014_MOESM3_ESM.docx]

**Supplemental Table 1:** Propensity score parameter list

| the variables used in calculating the propensity score | Female age, BMI, AFC, AMH, Infertility duration, Infertility type, Infertility factors, No. of transferred embryos and Embryo transfer day. |
| --- | --- |
| Propensity scoring algorithm | Logistic regression model |
| C-statistical | 0.6002 |
| Matching method | Greedy matching within specified caliper distances |
| Distance metric | 0.01 |
| Matching ratio | 1:1 |
| Use of replacement | Without replacement |
| Matching sample size | Long GnRH-a =964 cases Total:1928 |
|  | Depot GnRH-a=964cases |

BMI, body mass index; AFC, antral follicular count; AMH, anti-Müllerian hormone; GnRH-a, gonadotropin-releasing hormone agonist.
