# Supplementary material for: The follicular-phase depot GnRH agonist protocol results in a higher live birth rate without discernible differences in luteal function and child health versus the daily mid-luteal GnRH agonist protocol: a single-centre, retrospective, propensity score matched cohort study
Source: Reprod Biol Endocrinol. 2022 Sep 19;20:140. doi: 10.1186/s12958-022-01014-0 (PMC9483542; doi:10.1186/s12958-022-01014-0)
Supplement: Supplementary file 4 — Additional file 3: Supplemental Table 2. Comparison of the correlation between the two GnRH-a protocols and pregnancy outcomes using multivariable regression analysis and conditional logistic regression analysis before and after propensity score matching. [file 12958_2022_1014_MOESM4_ESM.docx]

**Supplemental Table** **2** Comparison of the correlation between the two GnRH-a protocols and pregnancy outcomes using multivariable regression analysis and conditional logistic regression analysis before and after propensity score matching.

| Exposure | Before propensity matching | | |  | After propensity matching | | |
| --- | --- | --- | --- | --- | --- | --- | --- |
|  | Non-adjusted | | Adjust I |  | PSM model | | Adjust II |
| Biochemical pregnancy rate |  |  | |  |  |  | |
| Long GnRH-a | 1.0 | 1.0 | |  | 1.0 | 1.0 | |
| Depot GnRH-a | 1.25 (1.08, 1.44) 0.0026 | 1.23 (1.03, 1.48) 0.0216 | |  | 1.24 (1.03, 1.51) 0.0254 | 1.24 (1.02, 1.51) 0.0273 | |
| Clinical pregnancy rate |  |  | |  |  |  | |
| Long GnRH-a | 1.0 | 1.0 | |  | 1.0 | 1.0 | |
| Depot GnRH-a | 1.23 (1.07, 1.41) 0.0037 | 1.20 (1.01, 1.43) 0.0393 | |  | 1.23 (1.03, 1.48) 0.0255 | 1.23 (1.02, 1.48) 0.0269 | |
| Live birth rate |  |  | |  |  |  | |
| Long GnRH-a | 1.0 | 1.0 | |  | 1.0 | 1.0 | |
| Depot GnRH-a | 1.20 (1.05, 1.37) 0.0089 | 1.15 (0.97, 1.36) 0.1015 | |  | 1.20 (1.00, 1.43) 0.0480 | 1.20 (1.00, 1.43) 0.0485 | |

Data was shown as OR (95%CI) *P* value.

Non-adjusted model adjusts for: None.
Adjust I model adjust for: female age, BMI, AFC, AMH, Infertility duration, Infertility type, Infertility factors, number of transferred embryos and Embryo transfer day.

Adjust II model adjust for: AMH.
